# Delayed MSC therapy enhances resolution of organized pneumonia induced by antibiotic resistant *Klebsiella pneumoniae* infection

**DOI:** 10.3389/fmed.2023.1132749

**Published:** 2023-07-03

**Authors:** Declan Byrnes, Claire Masterson, Jack Brady, Shahd Horie, Sean D. McCarthy, Hector Gonzalez, Daniel O’Toole, John Laffey

**Affiliations:** ^1^Anaesthesia, School of Medicine, Clinical Sciences Institute, University of Galway, Galway, Ireland; ^2^Regenerative Medicine Institute (REMEDI) at CÚRAM Centre for Research in Medical Devices, Biomedical Sciences Building, University of Galway, Galway, Ireland; ^3^Department of Anaesthesia, Galway University Hospitals, SAOLTA University Hospital Group, Galway, Ireland

**Keywords:** mesenchymal stromal cells, sepsis, immune tolerance, pneumonia, fibrosis, rodent animal models, peripheral blood mononuclear cells

## Abstract

**Introduction:**

Mesenchymal stromal cells (MSC) are a promising therapeutic for pneumonia-induced sepsis. Here we sought to determine the efficacy of delayed administration of naïve and activated bone marrow (BM), adipose (AD), and umbilical cord (UC) derived MSCs in organized antibiotic resistant *Klebsiella* pneumosepsis.

**Methods:**

Human BM-, AD-, and UC-MSCs were isolated and expanded and used either in the naïve state or following cytokine pre-activation. The effect of MSC tissue source and activation status was assessed first *in vitro*. Subsequent experiments assessed therapeutic potential as a delayed therapy at 48 h post infection of rodents with *Klebsiella pneumoniae*, with efficacy assessed at 120 h.

**Results:**

BM-, AD-, and UC-MSCs accelerated epithelial healing, increased phagocytosis, and reduced ROS-induced epithelial injury *in vitro*, with AD-MSCs less effective, and naïve MSCs more effective than pre-activated MSCs. Delayed MSC administration in pre-clinical organized *Klebsiella* pneumosepsis had no effect on physiologic indices, but enhanced resolution of structural lung injury. Delayed therapy with pre-activated MSCs reduced mRNA concentrations of fibrotic factors. Naïve MSC treatment reduced key circulating cell proportions and increased bacterial killing capacity in the lungs whereas pre-activated MSCs enhanced the phagocytic index of pulmonary white cells.

**Discussion:**

Delayed MSC therapy enhanced resolution of lung injury induced by antibiotic resistant *Klebsiella* infection and favorably modulated immune cell profile. Overall, AD-MSCs were less effective than either UC- or BM-MSCs, while naïve MSCs had a more favorable effect profile compared to pre-activated MSCs.

## 1. Introduction

Sepsis is defined as a dysfunctional immune response to infection, and it accounts for 20% of total mortality worldwide (1) with community-acquired bacterial pneumonia having the highest incidence rate among sepsis related infections (2). The most recent European Prevalence of Infection in Intensive Care study (EPIC III) shows that the most prevalent pathogens associated with increased mortality were hospital-acquired anti-biotic resistant strains of Enterococcus, Klebsiella, and Acinetobacter (3).

Sepsis can be divided into three phases, early, transition, and late sepsis, with each phase having distinct immunopathologies (4). Early-phase sepsis is characterized by a predominantly hyperinflammatory response, with increased cytokine production and inflammatory cell infiltration leading to increased capillary permeability, host tissue damage, end-organ damage, and a mortality rate of 10–15% (5). The transitionary phase is crucial in the resolution of sepsis as this is where the inflammation is becomes regulated and repair commences, with resulting changes in the immune cell profile including increased M2 macrophages (6), increased regulatory T cells (Tregs) (7), and reduction in natural killer (NK) cells and mature neutrophils (8). During this phase, the patient will either return to immune homeostasis, having controlled the infection with minimal cellular functional abnormalities, or, where infection persists, will enter what is known as late-phase sepsis; characterized by immunosuppression (9) and immune cell tolerance and exhaustion (10–12). It has been reported that up to 70% of all sepsis related deaths occur in the late phase of sepsis (13). Due to persistent exposure to injurious stimulation, these tolerant cells are unable to respond to further signals, therefore reducing the patient’s ability to combat secondary infections which occur in 39% of sepsis cases (14). In short, the later the phase of sepsis, the more difficult it is to combat using conventional methods.

Mesenchymal stromal cells (MSCs) are currently in clinical testing for several diseases and clinical syndromes due to their immunomodulatory capacity (15), pro-reparative functions (16), immune-evasive mechanisms (17), anti-microbial effects (18), and their efficacy in early-phase models of acute pneumonia sepsis [reviewed in (19)]. However, due to the pathology of sepsis and the necessary criteria, such as the SOFA score, that patients need to fall under to be considered ‘sepsis’, it can delay the administration of therapeutics which also contributes to the associated high mortality rate (20). The use of a freshly expanded and, or autologous MSC therapy could further contribute to the delay in administration. MSCs do have the potential to be administered as an off the shelf (cryopreserved) therapy from allogenic donors which would speed up the process (21), however a few factors such as determining an optimal tissue source of MSCs and dosing regimen, remains an open discussion in the field of regenerative medicine.

Bone marrow (BM) remains the most widely used MSC source because this was the first described source. Adipose (AD) and umbilical cord (UC) sources are becoming more widely studied with differing advantages of each source. All three cell types display surface markers characteristic of MSCs, as laid out by ISCT (22) with an exception being needed of the negative marker CD34 for adipose MSCs (23). The other standards they conform to are plastic adherence and multi-lineage differentiation potential.

This study aimed to identify the optimal tissue source of both naïve and cytokine pre-activated MSCs to enhance the resolution of late-phase organized antibiotic resistant Klebsiella pneumosepsis. The aim was to mimic the more clinically relevant situation when therapies are applied late in the evolution of the infection process.

## 2. Materials and methods

### 2.1. MSC culture and preparation

Bone marrow MSCs (BM-MSCs) and adipose MSCs from lipoaspirate (AD-MSC) were isolated from healthy volunteers at the Clinical Research Facility, University Hospital Galway, using standard isolation methods (22). Umbilical cord MSCs (UC-MSCs) were isolated from the perivascular tissues of healthy cords by Tissue Regeneration Therapeutics Ltd. (TRT Ltd., Toronto, Canada) and shipped at passages 1–3 (24, 25). The method of isolation, preparation and culture of these MSCs is detailed in online supplement.

MSCs were pre-activated at passage 3 using using cytomix (IL-1β (50 ng/mL), TNF-α (50 ng/mL) and IFN-ɣ (50 ng/mL)) (Immunotools Ltd., Friesoythe, Germany) for 24 h. MSCs in culture were freshly harvested, were washed twice in PBS before being typsinised and pelleted. Following two further washes in PBS, cells were counted and checked for viability using Trypan blue exclusion dye (Sigma) and administered within 1 h of harvest. Cell suspensions were over 85% viable in both naïve and pre-activated preparations. A viability of 70% was the lowest limit of viability that would be accepted.

Conditioned media (CM) was collected by replacing cytomix containing media with serum free media for a further 24 h. Cells were cryopreserved at passage 2 and characterized at passage 3 using flow cytometry (Supplementary Figure S1).

### 2.2. *In vitro* analyses of MSC function

#### 2.2.1. Nuclear factor-κB activation assay

An A549 pulmonary epithelial cell line incorporating a stably transfected κB-luciferase reporter construct (Thermo Fisher, Waltham, MA, United States) was grown in 96 well plates. Cell monolayers were injured using cytomix, or sham (vehicle) injury, then treated with CM from BM, UC, and AD-MSCs (with and without cytomix pre-activation), or vehicle control. Cells were assayed for luciferase content at 24 h using the OneGlo™ luciferase substrate assay (Promega, Madison, WI, United States) as an indicator of NF-κB activation and inflammation.

#### 2.2.2. Cell metabolic assessment of viability

MTT assays were performed to assess cell metabolic function as an index of viability using 3-(4, 5-dimethylthiazol-2-yl)-2,5-diphenyltetrazolium bromide (Sigma Aldrich Ltd., Wicklow, Ireland) reconstituted in culture medium (5 mg/mL) to evaluate cell viability and proliferation. Bronchial epithelial cell line (BEAS2B; ATCC) monolayers were subjected to oxidative injury using hydrogen peroxide (H_2_O_2_; Sigma) 8 mM, then treated with CM from BM, UC, and AD-MSCs (with and without cytomix pre-activation), or vehicle for 4 h. After treatment, cells were washed with PBS, followed by incubation with MTT reagent for 3 h at 37°C in a humidified cell culture incubator. Cells were lysed and the formazan solubilized using dimethyl sulfoxide (DMSO, Sigma) and absorbance readings were measured using the Varioskan^™^ Flash microplate reader (Thermo Fisher Ltd.) at 595 nm wavelength. The degree of cell viability was presented as a percentage relative to uninjured control.

#### 2.2.3. Inflammatory cytokine production and phagocytic index

The THP-1 monocyte-like cell line (ATCC) was used to generate a macrophage-like monolayer via 25 nM PMA exposure for 48 h with a subsequent 24 h of rest. Cells were exposed to either 100 ng/mL of *E. coli* lipopolysaccharide (LPS), or sham (vehicle), then treated with CM from BM, UC, and AD-MSCs (with and without cytomix pre-activation), or vehicle. TNF-α production was measured by ELISA (R&D Systems, UK). To measure the phagocytic capacity of these cells Zymosan A FITC BioParticles™ (Thermo Fisher Ltd.) were opsonised with human serum for 1 h before being added to the cells for 40 min. The cell monolayer was washed twice with DPBS before being fixed in 4% PFA for 10 min. The cells were washed twice and kept in DPBS until analysis using the Cytation 1 (BioTek Instruments, Inc.).

#### 2.2.4. Wound healing assay

Single linear wounds were created in confluent A549 cell monolayers with a 1,000 μL pipette tip in a 24-well plate, as previously described (26). Monolayers were randomized to incubation in CM from BM, UC, and AD-MSCs (with and without cytomix pre-activation), or vehicle, and the extent of wound closure measured 24 h later using the Cytation 1 (BioTek Instruments, Inc.).

#### 2.2.5. Neutrophil apoptosis assay

The HL-60 neutrophil-like cell line was exposed to 1.5% DMSO for 6 days to induce differentiation into polymorphonuclear (PMN) cells. Differentiated cells were exposed to 600 μM H_2_O_2_ for 4 h to induce apoptosis. Concurrently, cells were treated with CM from BM, UC, and AD-MSCs, with and without cytomix pre-activation, or vehicle. Levels of apoptosis were determined using FITC Annexin V and PI (Biolegend, San Diego, CA, United States) on the BD Accuri^™^ C6 Flow Cytometer and expressed as a percentage of total cells.

### 2.3. Ethics statement

All animal work was approved by the Animal Care Research Ethics Committee of the National University of Ireland, Galway and conducted under license from the Health Products Regulatory Authority, Ireland (Licence number AE19125/P067). Specific-pathogen-free adult male Sprague Dawley (CD) rats (Envigo, UK) weighing between 350 and 450 g were used in all experiments.

### 2.4. *Klebsiella pneumoniae*-induced lung injury

**IMAGE 1:**
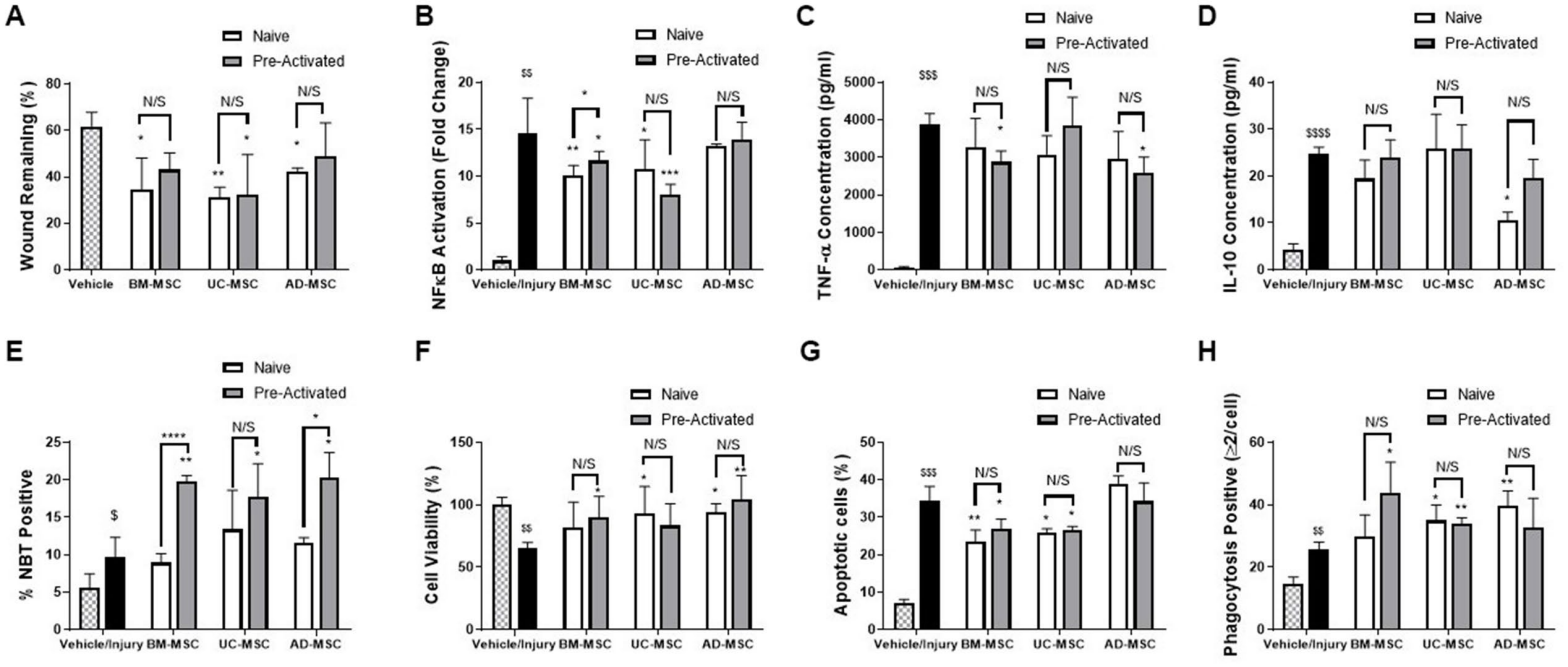
Schematic representation of the animal model of sepsis with male Sprague Dawley rats inoculated with a clinical strain of *Klebsiella Pneumoniae* intratracheally. After 48 h the animals are given either vehicle or one of the three sources of human MSCs IV. All pre- and post-mortem parameters are assessed at day 5 of the study.

In all groups, animals were administered pre-operative analgesia (Bupaq 0.03 mg/kg; Chanelle, Galway, Ireland) 1 h prior to anesthesia using isoflurane (Iso-Vet; Chanelle). The animals were then orotracheally intubated under direct vision with a 14G catheter (BD Insyte^®^; BD Biosciences). A bolus of 0.5 × 10^9^ CFU of clinically isolated, multidrug resistant, *K. pneumoniae* (Supplementary Table S1) in a 300 μL suspension was instilled followed by a bolus of air and the animals were allowed to recover from anesthesia as previously described (27) before proceeding to treatment at 48 h post inoculation. Results were collected 120 h post inoculation (Image 1).

### 2.5. Experimental design

#### 2.5.1. Series 1: naïve MSCs in long-term sepsis

To ascertain the optimal tissue source of MSCs in our long-term sepsis model, 48 animals were entered into series 1 and received 0.5 × 10^9^ CFU *K. pneumoniae* intratracheally at time zero. Forty-eight hours after pneumonia induction, animals received either (i) Vehicle, (ii) 1 × 10^7^ BM-MSC/kg, (iii) 1 × 10^7^ UC-MSC/kg, or (iv) 1 × 10^7^ AD-MSC/kg IV. Animals were monitored for a further 72 h before parameters were assessed at 120 h post pneumonia induction.

#### 2.5.2. Series 2: pre-activated MSCs in long term sepsis

To determine whether an additional effect of MSC pre-activation would be evident in our model, MSCs from the three tissue sources were exposed to cytomix as described for 24 h immediately prior to trypsinisation. Forty-eight hours after pneumonia induction, animals were randomized to receive (i) Vehicle, (ii) 1 × 10^7^ cytomix pre-activated BM-MSC/kg, (iii) 1 × 10^7^ cytomix pre-activated UC-MSC/kg, or (iv) 1 × 10^7^ cytomix pre-activated AD-MSC/kg IV (*n* = 12 per group). Animals were monitored for a further 72 h before parameters were assessed at 120 h post infection induction.

#### 2.5.3. Assessment of injury and recovery

At 120 h post pneumonia induction, animals were anesthetised with subcutaneous ketamine (75 mg.kg^−1^ Ketalar^™^; Pfizer, Cork, Ireland) and medetomidine (0.5 mg.kg^−1^ Dormidor^™^; Vetoquinol Ltd., Buckingham, UK). After confirmation of depth of anesthesia by paw pinch, IV access was obtained via tail vein using a 22G cannula (BD Insyte) and secured. Surgical tracheostomy was performed, using a 12G tracheostomy tube and intra-arterial access was gained by siting a 22G cannula in the right carotid artery for blood sampling and fluid administration. Anaesthesia was maintained with IV alfaxalone (2 mg.kg^−1^ Alfaxan^™^; Vetoquinol Ltd.) and paralysis with IV cisatracurium besylate (0.5 mg.kg^−1^ Tracrium^™^; GlaxoSmithKline PLC., London, UK). Protective mechanical ventilation (V_t_ 7 mL/kg, FiO_2_ 0.21) was commenced. Animals were ventilated for 15 min before static lung compliance was measured, following which inspired gas was changed to FiO_2_ 1.0 and ventilation proceeded for a further 7 min. Arterial blood gas analysis was performed after both ventilation stages as previously described (28).

#### 2.5.4. Bacterial load

At the end of the procedure, animals were sacrificed by exsanguination under anesthetic overdose and blood retained for PBMC isolation, CFU counts, and plasma collection. Bronchoalveolar lavage (BAL) fluids were collected from the lungs for cytokine profiles and bacterial load measurements. Blood and BAL were plated onto UTI agar plates (Fannin Ltd., Galway, Ireland) and incubated overnight at 37°C. Total colony number of *K. pneumoniae* were counted.

#### 2.5.5. Real-time PCR

Post BAL collection, lung tissue was minced and stored at −80°C for quantitative PCR. Following RNA extraction using Tri Reagent (Sigma), cDNA was synthesized with 1 μg of RNA using the Improm-II Reverse transcription kit (Promega) according to manufacturer’s instructions. qPCR was performed using a StepOnePlus^™^ Real-Time PCR System (Thermo Fisher) with a fast SYBR^™^ Green master mix (Thermo Fisher) and ultrapure water including the primers (Supplementary Table S2) at 10 pmol /well of a MicroAmp^®^ Fast Optical 96-well Reaction Plate (Applied Biosystems). Annealing temperature was set to 60°C with 40 cycles of amplification. RNA expression was normalized to GAPDH expression and analysed using the ΔΔCt method.

#### 2.5.6. Lung histology and stereology

The intact left lung was isolated and fixed using 4% PFA, and the extent of histologic lung damage was determined using quantitative stereological techniques as previously described (29) in addition to the SlideScan tool from GitHub.^1^

#### 2.5.7. Pulmonary white cell analysis

Total white cells and differential cell counts were performed on BAL fluid using cytospin columns and diff-quik staining (Fisher Sci, Ireland). The proportions of neutrophils, monocytes and other leukocytes were quantified. The phagocytic index of adherent BAL white cells was determined by visualizing ingestion of opsonized FITC-labeled zymosan particles (Thermo Fisher) using fluorescence microscopy (Cytataion 1, Biotek Ltd). Cells with >2 ingested particles were considered to be phagocytosing. The bacterial killing potential of the cells was determined by the production of ROS in the phagolysosome using the nitroblue tetrozolium (NBT) assay. Cells were exposed to 5 ng/mL NBT for 1 h and ROS production in the phagolysosome observed using light microscopy.

#### 2.5.8. Peripheral blood mononuclear cell isolation

Peripheral blood mononuclear cells were isolated from the whole blood of the experimental animals using histopaque 1077 (Sigma). Briefly, whole blood was diluted 1:1 with phosphate buffered saline (PBS) (Sigma-Aldrich) and layered over Histopaque-1077. The sample was then centrifuged at 400 × g for 30 min. The PBMC layer was removed and washed twice in Hanks Balanced Salt Solution (HBSS) (Sigma-Aldrich). After the final wash step the PBMCs were resuspended in PBS and viability assessed using Trypan Blue (Sigma-Aldrich). The PBMCs were then either stained for flow cytometry or plated at 8 × 10^4^ cells/well in a 96 well plate and exposed to 100 ng/mL LPS or vehicle for 24 h to determine their reactivity to an endotoxin stimulus. Media was collected and TNF-α and IL-6 secretion levels analysed by ELISA (R&D/Biotechne, Minneapolis, MN, United States) according to manufactures guidelines.

#### 2.5.9. Cell isolation from lung tissue

Rat lung tissue was minced and digested using collagenase IV (200 U/mL; Thermo Fisher Scientific) and DNAse 1 (200 U/mL; Millipore Sigma) at 37°C for 2 h with regular agitation. Single cell suspensions of digested lung were prepared by passing through a 40 μm cell strainer (Thermo Fisher Scientific) and centrifuging at 400 × g for 5 min. The cell pellet was then resuspended in 3 mL of 1X Red Cell Lysis Solution (Milteny Biotec) and incubated for 2 min at room temperature. The solution stopped by dilution with T-cell media and centrifuged at 400 × g for 5 min. The supernatant was discarded, and the cell pellet resuspended in 10 mL of PBS and counted.

#### 2.5.10. Antibody staining for flow cytometry

For flow cytometry, 10^6^ PBMCs or single cell lung digestions were stained with the relevant antibodies (Miltenyi Biotec and Thermo Fisher Scientific) and dead-cell exclusion dye DRAQ7 (BioLegend, San Diego, CA, United States) at their most optimal staining concentration predetermined by titration experiments. For FOXP3 staining, cells were fixed and permeabilised with FOXP3/Transcription Factor Staining Buffer Kit (Thermo Fisher Scientific) as per the manufacturer’s instructions. Briefly, single cell suspensions were prepared in azide/serum free PBS and washed twice. 1 μL of Ghost Dye Red 780 Viability Dye (Tonbo Biosciences, San Diego, CA, United States) was used to stain cells in 1 mL PBS. The PBMCs were then washed twice with flow cytometry stain buffer, stained, and incubated for 30 min on ice. The cells were then washed two more times and 1 mL of Fixation/Permeabilization buffer was added to each tube and pulse vortexed. The samples were incubated for at least 30 min. Next, 2 mL of 1X Permeabilization buffer was added, and samples were centrifuged for 5 min @ 400 × g at RT two times. The PBMCs were then blocked with an anti-CD32 antibody for 10 min before staining with FOXP3. The cells were incubated for 30 min at RT and protected from light before being washed two more times with 1X permeabilization buffer and finally resuspended in FACS buffer and analysed on the FACS Canto II Flow Cytometer (BD Biosciences). The cell population was gated on with doublet and dead cell exclusion occurring before data analysis (Supplementary Figure S2). Flow cytometry data was analysed using FlowJo analysis software v10.1 (BD Biosciences).

### 2.6. Statistical analysis

Statistical analysis of the data was performed using GraphPad Prism 8.0.1 software. The results are presented as the mean ± standard deviation (SD). Unpaired, two tailed student *T*-Tests (Mann–Whitney test) were used to compare relative changes in smaller datasets with a significance threshold of *p* < 0.01 to account for multiple comparisons within the sample. All data sets were subjected to Shapiro–Wilk test to test for normal distribution. Normally distributed data was analysed using parametric 1-way ANOVA with multiple comparisons and Dunnett’s statistical hypothesis testing. Data not normally distributed was analysed using non-parametric Kruskal-Wallis analysis correcting for multiple comparisons using Dunn’s test.

## 3. Results

### 3.1. Impact of MSC tissue source and activation status on mechanisms of action

**Figure 1:**
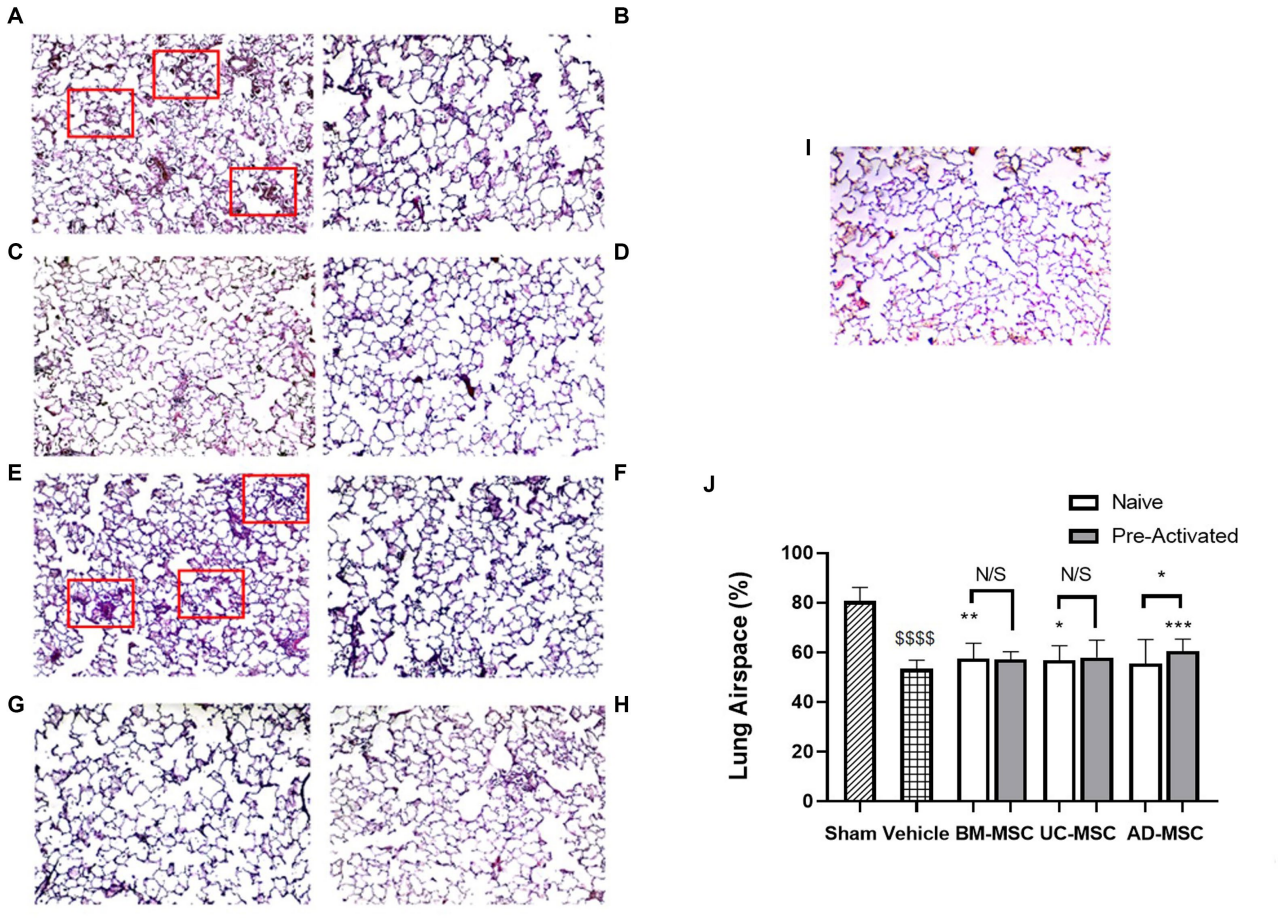
*In vitro* assessment of naïve and pre-activated MSCs from Bone marry, adipose tissue and umbilical cord. MSC-CM significantly reduced the wound size in A549 cell monolayers **(A)** compared to control and; decreased the inflammatory response to injury as shown by NF-κB activation in A549 cells **(B)**, MSC-CM significantly reduced the production of inflammatory TNF-α from endotoxin stimulated THP-1 monocyte./macrophage cells **(C)** Ad-MSC-CM, but not other MSC-CM, reduced change IL-10 secretion from endotoxin stimulated THP-1 cells **(D)**, while THP-1 NBT production was increased by pre-activated, but not naïve MSC-CM **(E)**. MSC-CM increased cell viability in BEAS2B cells when exposed to a ROS injury **(F)**. MSC-CM also significantly reduced the apoptosis induced by H2O2 exposure in HL-60s **(G)** and significantly increased the phagocytosis of THP-1 cells **(H)** (*N* = 4–6, Graphs representative of 3 independent experiments, bars represent mean + SD, *p ≤ 0.05 vs. injury, **p ≤ 0.01 vs. injury, ***p ≤ 0.001 vs. injury, ****p ≤ 0.0001 vs. injury. ^$^*p* ≤ 0.05 vs. vehicle, ^$$^*p* ≤ 0.01 vs. vehicle, ^$$$^*p* ≤ 0.001 vs. vehicle, ^$$$$^*p* ≤ 0.0001 vs. vehicle).

The three tissue sources of MSC-CM were compared using a panel of *in vitro* functional assays and demonstrated a varying profile of efficacy. Naïve BM-, UC-, and AD-MSC CM significantly improved wound closure of pulmonary epithelial cell monolayers subjected to scratch wound. Cytomix licensing reduced wound healing efficacy in all the MSC types (Figure 1A). Naïve BM- and UC-, but not AD-MSCs, were effective in reducing NF-κB mediated inflammation in pulmonary epithelial cells and cytomix pre-activation further reduced NF-κB in UC-MSCs only (Figure 1B). TNF-α secretion in endotoxin stimulated monocyte/macrophage immune cells was increased following injury, which was decreased in the presence of preactivated BM- and AD-MSCs (Figure 1C). Naïve AD-MSCs- but no other cell therapy—significantly reduced IL-10 secretion in monocyte/macrophage immune cells (Figure 1D). Monocyte/macrophage ROS production was significantly enhanced when exposed to pre-activated—but not naïve—MSC-CM compared to immune cells exposed to injury (Figure 1E). Naïve UC-MSCs, pre-activated BM-MSCs, and both naïve and pre-activated AD-MSCs improved lung epithelial cell viability after a severe H_2_O_2_ injury, while pre-activation did not further increase efficacy (Figure 1F). Both naïve and pre-activated BM- and UC-MSC CM significantly reduced the rate of apoptosis in neutrophil-like HL-60 cells when exposed to H_2_O_2_ and pre-activation did not significantly alter this (Figure 1G). Naïve UC- and AD- along with pre-activated BM- and UC-MSC CM significantly improved macrophage-like THP-1 phagocytosis (Figure 1H) and pre-activation did not significantly alter this. Supplementary Table S3 summarizes the performance of the different cells across this panel of *in vitro* assays.

### 3.2. MSC therapy enhances resolution of structural lung injury

**Figure 2:**
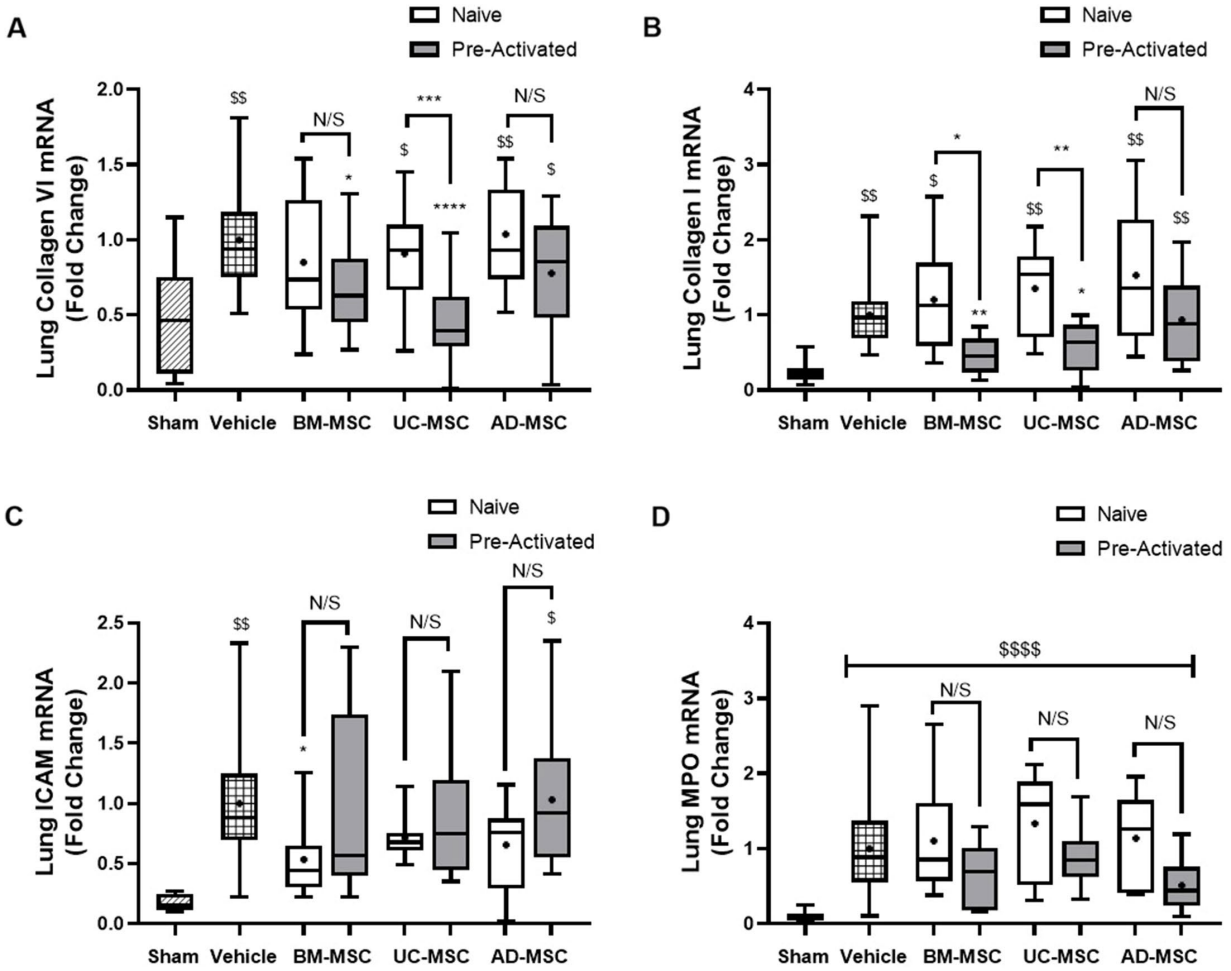
MSC therapy reduced Klebsiella pneumonia induced histological lung injury. At 5 days post klebsiella infection, there was evident loss of airspace due to alveolar thickening, atelectasis, and infiltration **(A)** compared to sham animals **(I)**. Administration of naïve BM- **(B)** or UC-MSCs **(C)**, but not naïve AD-MSCs **(D)** increased lung airspace compared to vehicle treated **(A)** animals. In contrast, preactivated pre-activated BM- **(F)**, UC- **(G)**, or AD- **(H)** MSCs did not restore airspace compared to vehicle treated **(E)** animals. A quantitative analysis is presented in **(J)**. Red boxes highlight areas of significant alveolar infiltration and injury in the vehicle groups (*N* = 10–12 animals per group, bars represent mean + SD, *p ≤ 0.05 vs. vehicle, **p ≤ 0.01 vs. vehicle, ***p ≤ 0.001 vs. vehicle, ****p ≤ 0.0001 vs. vehicle; ^$$$$^*p* ≤ 0.0001 vs. sham).

Klebsiella pneumonia infection resulted in increased alveolar wall thickening, cell infiltration to the alveolar space, and atelectasis, and reduced airspace fraction at 120 h (Figures 2A and 2E). The airspace fraction of the lung tissue was increased in animals treated with naïve BM- and UC-MSCs but not AD-MSCs compared to vehicle control (Figures 2A–D), as assessed quantitatively (Figure 2J). Conversely, pre-activated AD-MSCs—but not BM-MSCs or UC-MSCs restored lung airspace (Figures 2E–H). Quantitative analysis revealed the reduction in lung airspace resulting from *K. pneumoniae* infection, and the potential for naïve BM- and UC-MSCs, and activated AD-MSCs, to attenuate the lung airspace reduction (Figures 2J,I).

**Figure 3:**
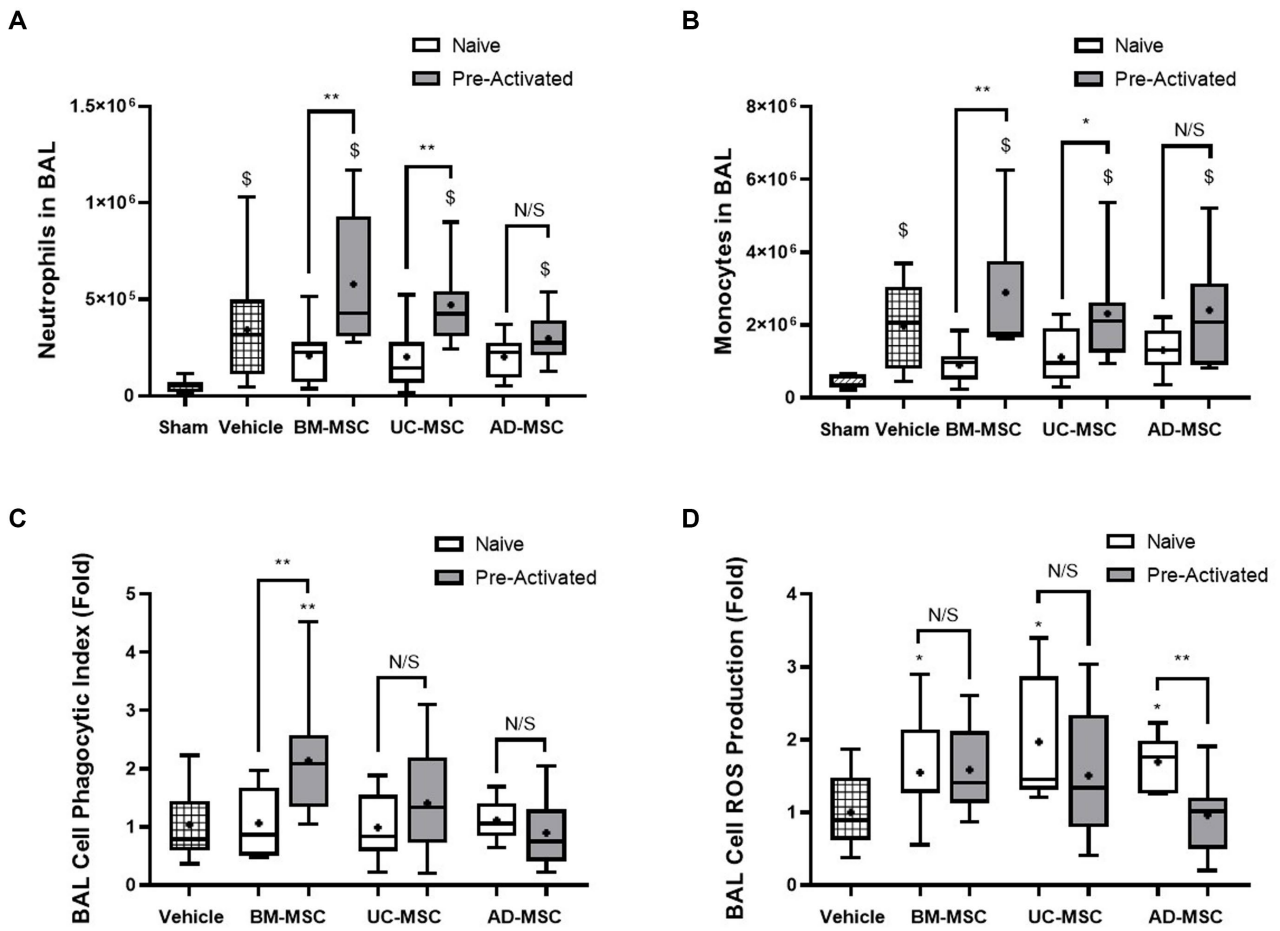
Effect of MSC therapy on Klebsiella pneumonia induced collagen, ICAM-1 and MPO mRNA concentrations. Naïve MSC administration at 48 h to long term models of pulmonary sepsis does not significantly lower the mRNA levels of collagen VI and I in the lung tissue **(A,B)** while pre-activated BM- and UC-MSCs significantly decreased the expression of collagen I & VI compared to vehicle **(A,B)**. Only naïve BM-MSCs significantly decrease the expression of ICAM-1 compared to vehicle control **(C)** and there were no changes in expression of ICAM compared to vehicle control for pre-activated MSC treatments **(C)**. MPO mRNA levels remained unchanged compared to vehicle control for naïve and pre-activated MSC treatment groups **(D)** (*N* = 4–6 per group, bars represent mean fold change from sham + SD, *p ≤ 0.05 vs. vehicle, **p ≤ 0.01 vs. vehicle, ***p ≤ 0.001 vs. vehicle, ****p ≤ 0.0001 vs. vehicle. ^$^*p* ≤ 0.05 vs. sham, ^$$^*p* ≤ 0.01 vs. sham, ^$$$^*p* ≤ 0.001 vs. sham, ^$$$$^*p* ≤ 0.0001 vs. sham).

Established Klebsiella pneumonia increased mRNA concentrations of Collagen I and VI and ICAM 1 and lung myeloperoxidase (Figure 3). Naïve MSCs did not alter mRNA levels of collagen I or collagen VI compared to vehicle (Figures 3A,B). In contrast, pre-activated UC-MSCs decreased collagen VI, and both pre-activated BM- and UC-MSCs decreased collagen I (Figures 3A,B). Naïve BM-MSCs significantly reduced the levels of ICAM mRNA compared to vehicle, whereas the other naïve or pre-activated MSCs had no effect (Figure 3C). There were no changes in myeloperoxidase (MPO) levels compared to vehicle treatment with any MSC type (Figure 3D). At 120 h post infection, the bacterial load in the lungs were low overall, and numerically lower with MSC therapy, but these differences were not statistically different (Supplementary Figure S3).

### 3.3. MSC therapy modulates the pulmonary and systemic immune response

**Figure 4:**
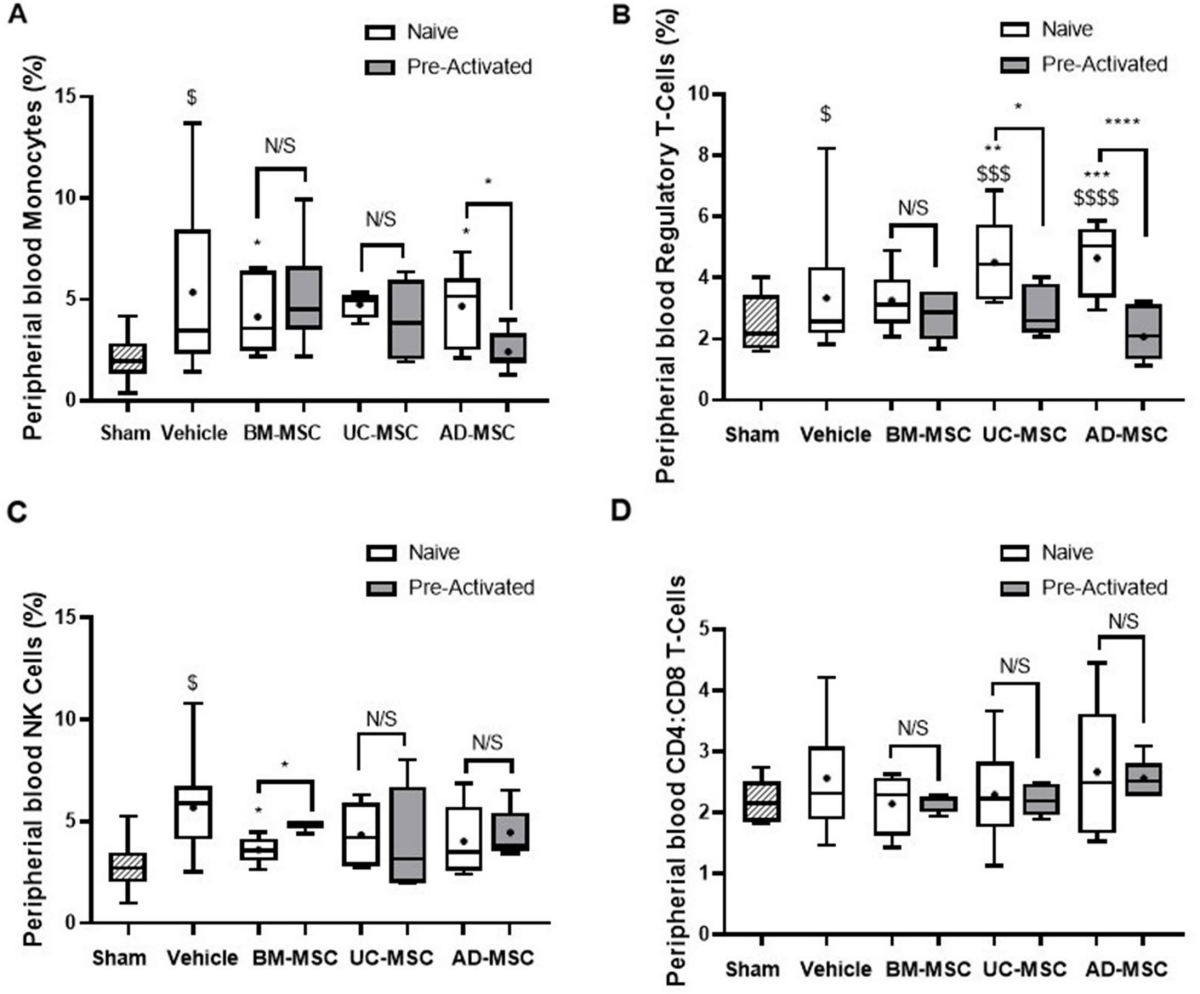
Effects of MSC therapy on Klebsiella pneumonia infection induced alveolar fluid neutrophils and monocytes. The total BAL neutrophil **(A)** and monocyte counts **(B)** were elevated compared to sham but not significantly different in naïve cell treated groups compared to vehicle control. Pre-activated MSC administration retained significantly higher levels of neutrophils **(A)** and monocytes **(B)** compared to sham. Following naïve MSC administration, ROS production in adherent BAL cells was significantly increased **(C)**, whereas the phagocytic index remained unchanged between groups. Pre-activated MSCs did not significantly affect ROS production **(C)**. The administration of pre-activated BM-MSCs resulted in increased phagocytic index of adherent BAL monocytes/macrophages compared to vehicle control **(D)** (*N* = 10–12 per group, box plot lines = median, error bars = min-max, bars represent mean + SD, *p ≤ 0.05 vs. vehicle, **p ≤ 0.01 vs. vehicle, ***p ≤ 0.001 vs. vehicle, ****p ≤ 0.0001 vs. vehicle. ^$^*p* ≤ 0.05 vs. sham, ^$$^*p* ≤ 0.01 vs. sham, ^$$$^*p* ≤ 0.001 vs. sham, ^$$$$^*p* ≤ 0.0001 vs. sham).

Established Klebsiella pneumonia increased neutrophils and monocytes in the BAL fluid in vehicle treated animals compared to sham (Figures 4A,B). In naïve MSC treated animals there was no significant increase in BAL neutrophils or monocytes compared to Sham. In contrast, pre-activated MSCs significantly increased BAL neutrophils and monocytes compared to Sham. There was no significant change between vehicle and MSC treated animals for the number of neutrophil and monocytes in the BAL fluid (Figures 4A,B). Adherent BAL cells isolated from naïve MSC-treated animals had improved ROS production but did not have an improved phagocytic index compared to vehicle (Figures 4C,D). In contrast, BAL cell ROS production was not enhanced after pre-activated MSC treatment (Figure 4C) but animals treated with pre-activated BM-MSCs MSCs had an improved phagocytic index compared to vehicle (Figure 4D).

**Figure 5:**
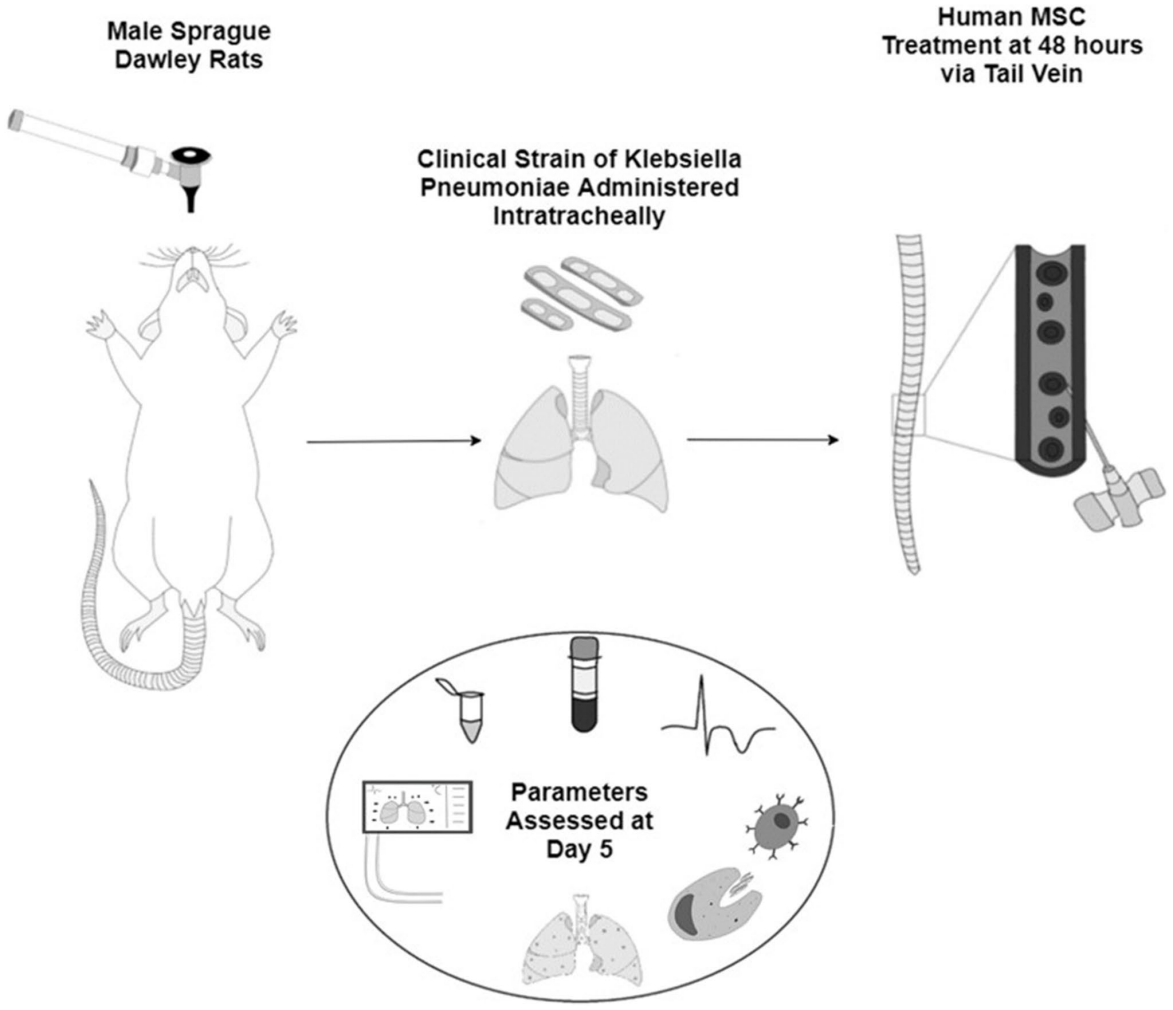
Effects of MSC therapy partially altered peripheral blood white cell profile. Naïve MSC therapy attenuated the increase in monocytes seen in the vehicle treated animals, this was not seen in the pre-activated MSC treated animals **(A)**. There was a significant increase in suppressive CD4+ T-regs in the naïve vehicle group compared to sham that was not seen in the pre-activated treatment groups **(B)**. MSCs therapy does not modulate NK **(C)** or CD4^+^:CD8^+^ T cell ratios **(D)** in the peripheral blood mononuclear cells (*N* = 5–7 per group, box plot lines = median, error bars = min-max, bars represent mean + SD, *p ≤ 0.05 vs. vehicle, **p ≤ 0.01 vs. vehicle, ***p ≤ 0.001 vs. vehicle, ****p ≤ 0.0001 vs. vehicle. ^$^*p* ≤ 0.05 vs. sham, ^$$^*p* ≤ 0.01 vs. sham, ^$$$^*p* ≤ 0.001 vs. sham, ^$$$$^*p* ≤ 0.0001 vs. sham).

*Klebsiella pneumonia* increased the percentage of monocytes in the peripheral blood of vehicle treated animals, and this increase was attenuated by naïve BM- and AD-MSC cell treatment (Figure 5A). Naïve UC- and AD-MSCs—but not the other cell types—significantly increased the proportion of Tregs compared to vehicle treated and sham animals (Figure 5B). The increase in NK cell proportions in vehicle treated animals was attenuated by naïve and pre-activated BM-MSC therapy, but not by the other cell types (Figure 5C). There were no significant changes in the classical and non-classical subtypes of monocytes across all treatment groups (data not shown), nor in the ratio of CD4^+^ helper and CD8^+^ cytotoxic T cell populations among all animal groups (Figure 5D).

## 4. Discussion

Mesenchymal stromal cell therapy for critical illnesses such as acute respiratory distress syndrome and sepsis has progressed from positive preclinical experiments to clinical trials using a variety of human tissue sources, isolation techniques and expansion protocols. These pre-clinical investigations have largely focused on on early-phase sepsis with MSC administration typically occurring pre-symptomatic or early in the disease time course (19).

However, patients generally present later in the course of their severe infection, and so studies that model these later phases are required.

The optimal MSC source for clinical translation also remains unclear. While BM is the most used and therefore the most understood MSC, they only comprise 0.001–0.01% of bone marrow cells and tend to undergo early senescence, and bone marrow harvest is an invasive procedure (30). AD-MSCs are also accessible from healthy donors, typically requiring liposuction, which is also an invasive procedure. However, AD-MSCs occur in much higher frequencies, up to 500 times more than BM (31) and proliferate longer than BM (30). UC-MSCs are acquired from the most immature source of the three allowing for greater proliferation compared to the other sources (32), reduced telomere shortening, and less chances of being environmentally altered due to previous infections (33). Previous reports, though limited, suggest that the immunosuppressive properties and proliferative capacity of AD- and UC-MSCs may be better than BM-MSCs (34). Molecular mechanisms underlying enhanced immunosuppressive effects may include enhanced secretion of the cytokines IL-10 and TGFβ (35). However, given the inconsistencies in the literature we developed a series of *in vitro* assays to directly compare these three MSC cell sources.

Our initial *in vitro* assessment gave great promise to the effects of MSCs on tissue healing and innate cell functionality, demonstrating the varying profile of MSC functions depending on their tissue source and their naïve vs. cytokine pre-activated state. We pre-activated MSCs with a cytokine mix, because this has previously proven an effective enhancer of therapy in preclinical models (36). Overall, our findings in the *in vitro* assays suggest that BM- and UC-MSC performed comparably in the majority of assays, out-performing AD-MSCs, while preactivated BM-MSCs performed best.

To address these translational gaps, we developed a later phase pneumosepsis model utilizing an antibiotic resistant bacterial pathogen with a relatively extended duration of infection and injury to identify the optimal tissue source of both naïve and cytokine pre-activated MSCs to aid resolution of this more established pneumosepsis. We wished to determine whether the previously demonstrated protective role of MSCs on lung function during earlier phases of lung infection, as shown by Masterson et al. (27), would translate to this later phase pneumosepsis model.

Our findings in the later phase Klebsiella pneumonia infection model demonstrate the greater potential for delayed therapy with naïve MSC therapy, compared to pre-activated MSC therapy, to facilitate restoration of lung structure, increasing lung airspace. Our pre-clinical studies further supported our *in vitro* findings, with AD-MSCs generally less effective than either UC- or BM-MSCs. Overall, the MSCs were less effective than when administered in earlier phase pneumonia models (27), which is perhaps not unexpected. Studies have shown that MSCs can preserve or restore the epithelial and endothelial barrier in the lungs by reducing inflammation, improving tissue healing, increasing local cell survival, and enhancing autophagy (37). This may have occurred here however; the majority of physiological parameters had returned to healthy control levels at this late phase of infection, and so an effect after the administration of MSCs cannot be seen.

Further *ex vivo* analysis demonstrated an increased ROS production in BAL macrophage/monocyte cells, in animals administered any of the naïve MSC types, as previously demonstrated (38). This contrasts with our *in vitro* experiments where naïve MSCs showed only slight increases in ROS production. BAL cell phagocytosis, previously shown to be enhanced in inflammatory ARDS (39), was not affected by naïve MSC therapy during the late phase. Again, this contrasted with *in vitro* exposure of various immune and lung cell types to naïve MSC-derived medium, where phagocytosis was increased, epithelial wound closure accelerated, and cell protection observed during hydrogen peroxide injury. The fact that the *in vitro* studies were conducted using the MSC secretome only, while the *in vivo* studies used the whole cells, and the fact the *in vitro* models are of human (rather than animal) origin, may at least partially may explain these differences.

In other analyses, peripheral blood monocytes were decreased with naïve BM- and AD-MSC therapy, while the increase in NK cells was attenuated by naïve and pre-activated BM-MSC therapies. In addition, Tregs were increased with naïve UC- and AD-MSCs, but not with the other cell types. However, as these proportion changes were small and there was no translation to an effect in *in vivo* physiology, it is difficult to ascertain the significance of this. The effect of the injury on circulating PBMC proportions was lost when using pre-activated cells. Again, this would point to the current standard MSC therapeutics likely being ineffective in later phase sepsis, suggesting the focus should remain on delivery of MSC therapy as early as possible in the disease time course to prevent both mortality in early phase and progression to later phase sepsis.

In healthy lungs there is a balance between degradation and synthesis of collagen that is disrupted during fibrosis. Gene expression analysis also revealed differences between animals receiving naïve and pre-activated MSCs. The levels of mRNA which would indicate the development or progression of fibrosis and inflammation were analysed in the lung tissues of animals in all groups. The levels would indicate that while there was not a substantial amount of collagen mRNA, it was significantly increased compared to sham in the case of collagen I and VI. Expression of both collagen types was decreased in animals receiving pre-activated BM- and UC-MSCs, but not naïve MSCs from any source. Levels of ICAM were reduced in tissues from animals treated with naïve BM-MSCs and largely unchanged in groups administered other cell types and pre-activated cells. While pro-collagen has to be cleaved before it is incorporated into lung tissue (40), these findings suggest that the potential for fibrosis may be reduced by treatment with pre-activated BM- and UC- MSCs.

Given the trend for loss of effect after pre-activation in this study, and its promise in other studies would indicate that this may not be the optimal pre-activation strategy for this stage of pulmonary sepsis or for late administration time-points. Cumulatively, BM-MSCs without pre-activation had the greatest impact overall, however due to a lack of translation in physiological parameters, further investigation will be needed. An alternative animal model, different dosing regimens, and another pre-activation strategy should be considered.

## 5. Conclusion

Our findings in the later phase Klebsiella pneumonia infection model demonstrate the potential for delayed therapy with naïve—but not pre-activated—MSC therapy to facilitate restoration of lung structure, increasing lung airspace. While the effects of MSC were less marked than those seen in earlier phase pneumonia, this is not unexpected given the established nature of this injury. Overall, our findings in the *in vitro* assays, and in our model of delayed MSC treatment of Klebsiella induced pneumonia suggested that AD-MSCs were less effective than either UC- or BM-MSCs. In addition, the naïve MSCs appear to have a more favorable profile of effect when compared to the pre-activated MSCs. Taken together, these are important insights into the likely effectiveness of this advanced therapeutic medicinal product and will inform future drug development and clinical trial inclusion parameters.

## Data availability statement

The raw data supporting the conclusions of this article will be made available by the authors, without undue reservation.

## Ethics statement

The animal study was reviewed and approved by Animal Care Research Ethics Committee of the National University of Ireland, Galway.

## Author contributions

DO’T and JL: conceptualization, supervision, project administration, and funding acquisition. CM and SH: methodology. CM: validation. DB and CM: formal analysis, writing—original draft preparation. CM, DB, JB, and SH: investigation and data curation. DO’T, CM, and JL: writing—review and editing. All authors contributed to the article and approved the submitted version.

## Funding

This research was funded by Science Foundation Ireland, grant number Science Foundation Ireland award 16-FRL-3845 (JL) and Health Research Board Ireland award ILP-POR-2017-024 (DO’T).

## Conflict of interest

The authors declare that the research was conducted in the absence of any commercial or financial relationships that could be construed as a potential conflict of interest.

## Publisher’s note

All claims expressed in this article are solely those of the authors and do not necessarily represent those of their affiliated organizations, or those of the publisher, the editors and the reviewers. Any product that may be evaluated in this article, or claim that may be made by its manufacturer, is not guaranteed or endorsed by the publisher.

## Footnotes

^1^ https://github.com/CMasterson/ScanSlide2.git
