# Supplementary material for: Delayed MSC therapy enhances resolution of organized pneumonia induced by antibiotic resistant Klebsiella pneumoniae infection
Source: Front Med (Lausanne). 2023 Jul 3;10:1132749. doi: 10.3389/fmed.2023.1132749 (PMC10352103; doi:10.3389/fmed.2023.1132749)
Supplement: Supplementary file 1 [file Data_Sheet_1.docx]

Supplementary Material

Delayed MSC therapy enhances resolution of organized pneumonia induced by antibiotic resistant *Klebsiella pneumoniae* infection.

Declan Byrnes^1,2^†, Claire Masterson^1,2^†, Jack Brady^1,2^, Shahd Horie^1,2^, Sean D. McCarthy^1,2^, Hector Gonzalez^1,2^, Daniel O’Toole^1,2‡^, John Laffey^1,2,3‡*^

^1^ Anaesthesia, School of Medicine, Clinical Sciences Institute, University of Galway, Galway, Ireland

^2^ Regenerative Medicine Institute (REMEDI) at CÚRAM Centre for Research in Medical Devices, Biomedical Sciences Building, University of Galway, Galway, Ireland

^3^ Department of Anaesthesia, Galway University Hospitals, SAOLTA University Hospital Group, Galway, Ireland.

*Correspondence: Prof John G. Laffey, Anaesthesia, School of Medicine, University of Galway, Galway, Ireland; john.laffey@nuigalway.ie. Tel: +353 (0)91 495662

† These authors contributed equally to this work and share first authorship

^‡^ These authors contributed equally to this work and share senior authorship

# Supplementary Methodology

## MSC culture and preparation

***Bone marrow MSCs:*** Bone marrow MSCs (BM-MSCs): Bone marrow MSCs (BM-MSCs) were isolated from healthy volunteers using standard isolation methods [1]. In brief, 5x10^4^ mononuclear cells/cm^2^ in complete human MSC medium; α-MEM (Gibco 32561-029) supplemented with 10% FBS, 1% penicillin and streptomycin, and 25 µg/µL FGF-2 (ImmunoTools GmbH, Friesoythe, Germany), per T-175 flask. Four days after plating the cells, the monolayer was washed with DPBS (Thermo Fisher) and fresh medium was replaced with further media changes occurring every 2-3 days until cells were 70-80% confluent. Cell monolayers were trypsanised (Sigma-Aldrich), washed, and either replated at 5x10^5^ per T175 or cryopreserved in 90% FBS and 10% DMSO.

***Adipose MSCs:*** Adipose MSCs (AD-MSCs) were isolated from lipoaspirate as previously described [2]. In brief, the sample was allowed to stand in order to separate into three distinctive layers, an upper layer of oil, middle layer of adipose tissue, and a bottom layer of liquid infranatant containing contaminating cells. Both the upper and bottom layer were discarded while the middle layer was washed and subsequently digested with an equal volume of 0.1% collagenase IV (Thermo Fisher). This was done at 37°C for 2 hours with regular agitation until the fat solids disintegrated. Collagenase digestion was inactivated by the addition complete human MSC media, washed with DPBS, and subsequently filtered through a 100 µm cell strainer (Fisher Scientific, Massachusetts, USA). Cells were either cryopreserved or plated at a density of 5x10^5^ per T-175.

***Umbilical cord MSCs:*** UC-MSCs were isolated from the perivascular tissues of healthy cords according to a protocol generated and used by Tissue regeneration therapeutics Ltd. [3, 4]. Briefly, healthy umbilical cords were collected from full term births and the three individual blood vessels separated longitudinally from the rest of the tissue. The perivascular tissue was digested using 100U/mL collagenase type I and 0.01 U/mL hyaluronidase at 37^o^C for 3-5 hours. The supernatant was centrifuged at 285 x g for 10 minutes and the cells washed, counted, and plated. Cells were sub-cultured, cryopreserved, and shipped in liquid nitrogen vapour phase.

***MSC Activation:*** MSCs were pre-activated at passage 3 using using cytomix (IL-1β (50ng/mL), TNF-α (50ng/mL) and IFN-ɣ (50ng/mL) (Immunotools Ltd., Friesoythe, Germany) for 24 hours. Cells were washed twice in PBS before being typsinised and pelleted. Following two further washes in PBS, cells were counted and checked for viability using Trypan blue exclusion dye (Sigma) prior to administration. Cell suspensions were over 85% viable in both naïve and pre-activated preparations. A viability of 70% was the lowest limit of viability that would be accepted.

***Conditioned Media:*** Conditioned media (CM) was collected by replacing cytomix containing media with serum free media for a further 24 hours. Cells were cryopreserved at passage 2 and characterised at passage 3 using flow cytometry (Supplementary, Figure S1).

All cells were freshly harvested from cultures, and were washed, typsinised, pelleted, then counted and checked for viability, and administered all within 1 hour of harvest.

# Supplementary Figures and Tables

## Supplementary Tables

|  | Sample No. | *K. pneumoniae* |
| --- | --- | --- |
| ≥19-<19  Cefoxitin | **FOX** | 22 |
| ≥21-<21  Cefpodoxime | **CPD** | **6** |
| ≥5 difference between this and CPD  CPD/Clavulanic acid | **CPD/CV** | **17** |
| ≥22-<16  Meropenem | **MEM** | 32 |
| ≥14-<14  Ampicillin | **AMP** | **6** |
| ≥17-<17  Chloramphenicol | **C** | 25 |
| ≥18-≤13  Kanamycin | **K** | **13** |
| ≥17-<17  Gentamycin | **CN** | **7** |
| ≥15-<15  Trimethoprim | **W** | **6** |
| ≥25-<22  Ciprofloxacin | **CIP** | **15** |
| ≥15-≤11  Tetracycline | **TET** | 20 |
| ≥19-≤13  Nalidixic acid | **NA** | 15 |
| ≥22-<19  Ceftazidime | **CAZ** | **9** |
| ≥15-≤11  Streptomycin | **S** | **6** |
| ≥20-<17  Cefotaxime | **CTX** | **6** |

**Supplementary Table 1.** Microbial characterization of our clinically isolated *Klebsiella pneumoniae* strain.

| Target mRNA | Forward Primer (5’-3’) | Reverse Primer (5’-3’) |
| --- | --- | --- |
| GAPDH | TGCCAGCCTCGTCTCATAG | ACTGTGCCGTTGAACTTGC |
| Collagen I | TCATCGAATACAAAACCACCA | GCAGGGCCAATGTCCATT |
| Collagen VI | GGGACACACGTCTTCAGGTT | CCATGACTGATTGTTGTTGGG |
| ICAM | AGAAGCCTTCCTGCCTCG | TTGGGGTCCCCATCCACT |
| MPO | GCGATAGGTTTTGGTGGGAG | AGCTCACAAAGTCTCGGGG |

**Supplemental Table 2 –** Rat forward and reverse primers for RT-PCR analysis using Fast SYBR Green master mix.

| Assay | Cell Type | | | | | |
| --- | --- | --- | --- | --- | --- | --- |
|  | BM-MSC  Naive | BM-MSC  Preactivated | UC-MSC  Naive | UC-MSC  Preactivated | Ad-MSC  Naive | Ad-MSC  Preactivated |
| NF-κB Activation | **✓** | **✓** | **✓** | **✓** |  |  |
| Scratch Wound Assay | **✓** |  | **✓** | **✓** | **✓** |  |
| Cell Viability |  | **✓** | **✓** |  | **✓** | **✓** |
| Apoptosis in Neutrophil like cells | **✓** | **✓** | **✓** | **✓** |  |  |
| Sequestering TNF-α |  | **✓** |  |  |  | **✓** |
| Enhancing IL-10 secretion |  |  |  |  |  |  |
| Phagocytosis in Macrophage like cells |  | **✓** | **✓** | **✓** | **✓** |  |
| NBT in Macrophage like cells |  | **✓** |  | **✓** |  | **✓** |
| **Total** | **3** | **6** | **5** | **5** | **3** | **3** |

**Supplemental Table 3 –** Performance of each cell type – both naïve and preactivated I each of the in vitro assays. A ‘tick’ means the cell conditioned medium had a positive effect in that assay.

## Supplementary Figures


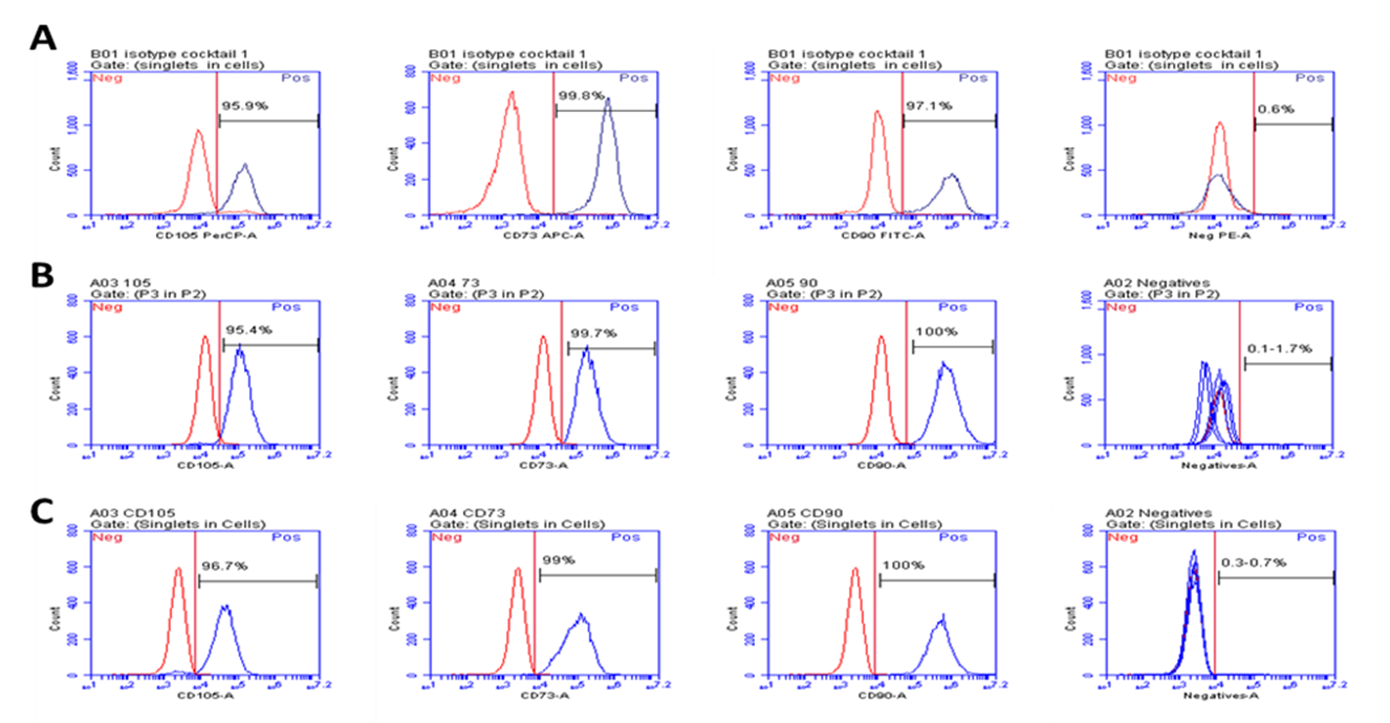


**Supplementary Figure 1.** Flow cytometry characterization of BM-MSC (A), UC-MSC (B), and AD-MSC (C) at the end of passage 3.


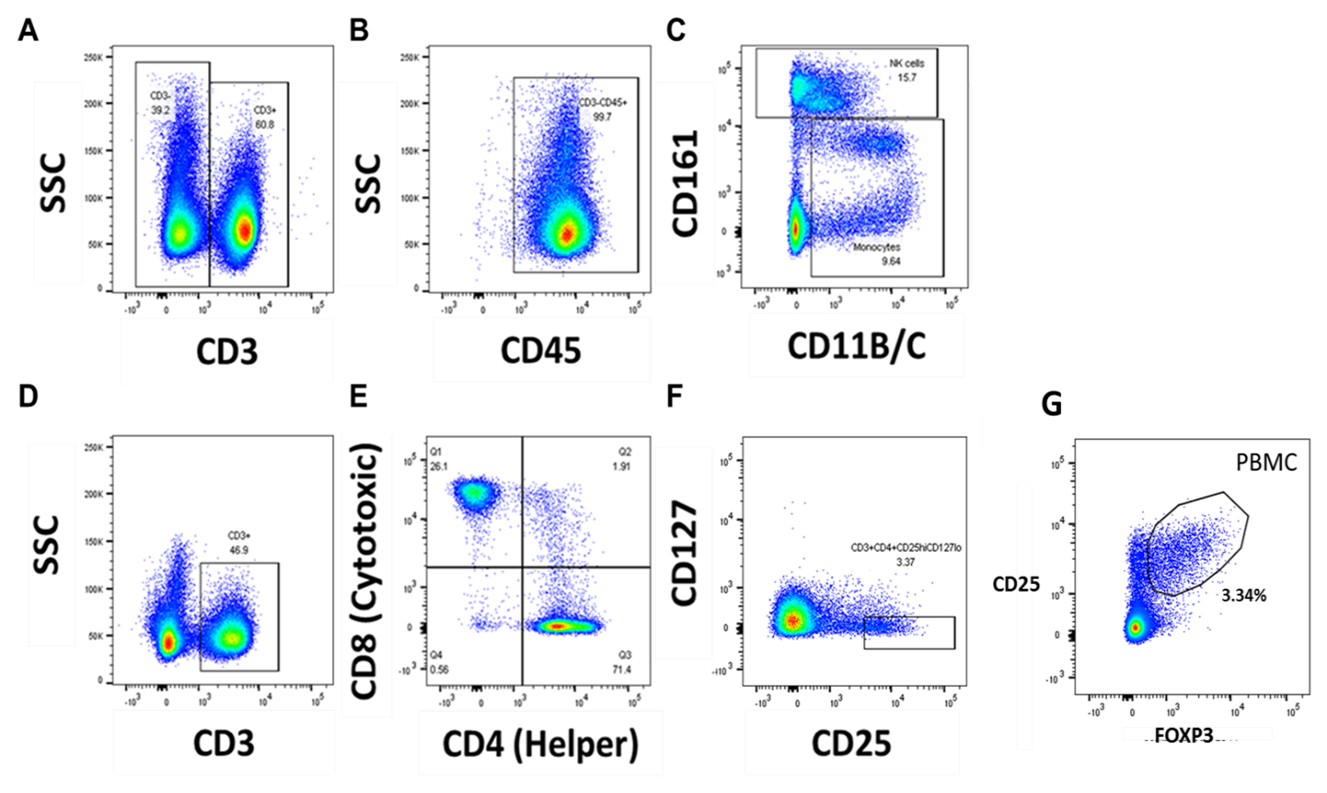


**Supplementary Figure 2.** Flow cytometry gating strategy for cell identification


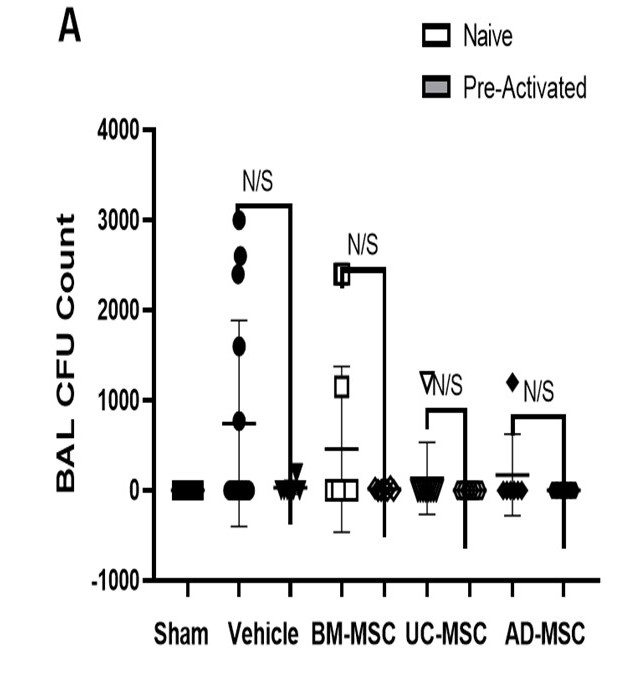


**Supplementary Figure 3.** BAL bacterial load shows no difference between sham, vehicle, and treatment groups (N=5-7 per group,

**References**

1. Dominici, M., et al., *Minimal criteria for defining multipotent mesenchymal stromal cells. The International Society for Cellular Therapy position statement.* Cytotherapy, 2006. **8**(4): p. 315-7.

2. Zhu, M., et al., *Manual isolation of adipose-derived stem cells from human lipoaspirates.* J Vis Exp, 2013(79): p. e50585.

3. Sarugaser, R., et al., *Human umbilical cord perivascular (HUCPV) cells: a source of mesenchymal progenitors.* Stem Cells, 2005. **23**(2): p. 220-9.

4. Sarugaser, R., et al., *Isolation, propagation, and characterization of human umbilical cord perivascular cells (HUCPVCs).* Methods Mol Biol, 2009. **482**: p. 269-79.
